# Role of albumin infusion in cirrhosis-associated complications

**DOI:** 10.1007/s10238-024-01315-1

**Published:** 2024-03-29

**Authors:** Mohamed A. Heybe, Kosha J. Mehta

**Affiliations:** 1 GKT School of Medical Education, Faculty of Life Sciences and Medicine, King’s College London, London, UK; 2 Centre for Education, Faculty of Life Sciences and Medicine, King’s College London, London, UK

**Keywords:** Cirrhosis, Chronic liver disease, Albumin, Ascites, Hepatorenal syndrome, Plasma expansion, Circulatory dysfunction

## Abstract

Cirrhosis is an advanced-stage liver disease that occurs due to persistent physiological insults such as excessive alcohol consumption, infections, or toxicity. It is characterised by scar tissue formation, portal hypertension, and ascites (accumulation of fluid in the abdominal cavity) in decompensated cirrhosis. This review evaluates how albumin infusion ameliorates cirrhosis-associated complications. Since albumin is an oncotic plasma protein, albumin infusion allows movement of water into the intravascular space, aids with fluid resuscitation, and thereby contributes to resolving cirrhosis-induced hypovolemia (loss of extracellular fluid) seen in ascites. Thus, albumin infusion helps prevent paracentesis-induced circulatory dysfunction, a complication that occurs when treating ascites. When cirrhosis advances, other complications such as spontaneous bacterial peritonitis and hepatorenal syndrome can manifest. Infused albumin helps mitigate these by exhibiting plasma expansion, antioxidant, and anti-inflammatory functions. In hepatic encephalopathy, albumin infusion is thought to improve cognitive function by reducing ammonia concentration in blood and thereby tackle cirrhosis-induced hepatocyte malfunction in ammonia clearance. Infused albumin can also exhibit protective effects by binding to the cirrhosis-induced proinflammatory cytokines TNFα and IL6. While albumin administration has shown to prolong overall survival of cirrhotic patients with ascites in the ANSWER trial, the ATTIRE and MACHT trials have shown either no effect or limitations such as development of pulmonary oedema and multiorgan failure.  Thus, albumin infusion is not a generic treatment option for all cirrhosis patients. Interestingly, cirrhosis-induced structural alterations in native albumin (which lead to formation of different albumin isoforms) can be used as prognostic biomarkers because specific albumin isoforms indicate certain complications of decompensated cirrhosis.

## Introduction

### Physiological role of albumin

Albumin is a major protein found in the blood, primarily synthesised by the liver. It represents half of the total protein content of plasma (3.5 g/dL to 5 g/dL) at a concentration of ≈40 mg/ml [1]. Albumin’s primary role is to regulate oncotic pressure (i.e. osmotic pressure dictated by colloids) [2]. It exerts a high oncotic pressure within blood vessels, attracting cations such as sodium (Nicholson et al., 2000), and it can do so because of its large molecular weight and strong negative charge. Due to this electrostatic attraction, water molecules are drawn passively into the intravascular compartment. This influx of water, in turn, aids in expanding the plasma volume, ensuring the maintenance of proper fluid balance within the circulatory system [1]. These attributes of albumin help to regulate fluid balance in the body.

An abnormal deficiency of albumin results in disruption of the delicate balance between hydrostatic pressure and oncotic pressure. This leads to reduced oncotic pressure, which in turn causes the movement of water from the intravascular compartment into the surrounding tissues [1]. Consequently, the regulatory effect of albumin on oncotic pressure is compromised, leading to the development of oedema [3].

### Background on cirrhosis and its complications

Cirrhosis is an advanced stage of chronic liver disease. It occurs due to persistent liver injuries by viral infections (e.g. hepatitis B and C viruses) or chronic alcohol consumption, and it can also be observed in non-alcoholic fatty liver disease (NAFLD), and the iron overload condition haemochromatosis [4]. Cirrhosis is characterised by several features including the presence of excessive scar tissue, i.e. fibrosis of liver parenchyma, formation of hepatocellular nodules [5] and portal hypertension (increased pressure in the portal venous system), that eventually lead to the development of varices and ascites (explained below). Cirrhosis tends to progress from an asymptomatic phase of compensated cirrhosis (where there is a gradual decline in liver function) to a symptomatic phase of decompensated cirrhosis where there is a marked deterioration in liver function [6]. Progression to decompensated cirrhosis often results in hospitalisation of patients due to worsening portal hypertension, systemic inflammation and liver failure [7].

One of the complications of decompensated liver cirrhosis is ascites, in which there is an accumulation of fluid in the peritoneal cavity [8]. It occurs in about 50% of patients with cirrhosis within 10 years of diagnosis [9]. The development of ascites appears to be stepwise. Initially, there is a marked increase in cirrhosis-induced intrahepatic resistance to portal blood flow. This results in increased portal pressure and portal hypertension [10] that causes a leak of fluid from the intravascular compartment into the peritoneal cavity. This hypoperfusion causes the renin angiotensin-aldosterone system (RAAS) to be activated leading to fluid retention. The release of aldosterone also causes sodium and fluid reabsorption [8]. This then promotes further fluid to be released into the peritoneal cavity, which increases the volume of ascites [8].

Decompensated cirrhosis is generally irreversible as long as the causative factor (such as alcohol and hepatitis virus) persists. Currently, the only curative resolution is liver resection or transplantation. Hence, the approach for managing cirrhosis is to identify its cause and prevent its progression to the decompensated stage, and thereby also decrease predisposition to hepatocellular carcinoma because cirrhosis is a risk factor for hepatocellular carcinoma development [11].

In principle, the aim of cirrhosis treatment and/or management is to decelerate the progression of liver damage by alleviating portal hypertension, and provide symptomatic relief to patients [5, 12].

### Overview of the role of albumin infusion in cirrhosis management

**Fig. 1:**
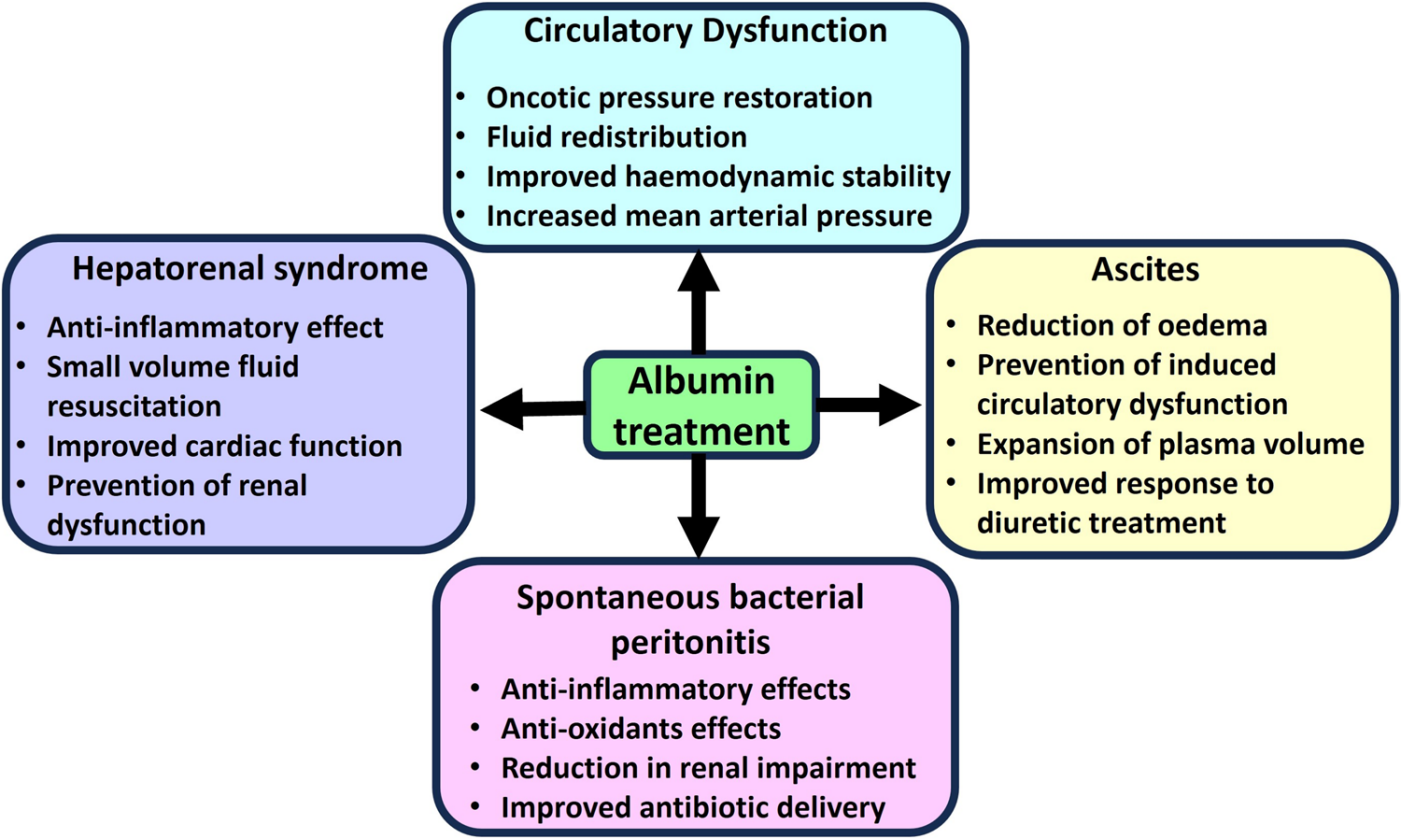
**The role of albumin in treating complications of cirrhosis.** Circulatory dysfunction, hepatorenal syndrome, ascites and spontaneous bacterial peritonitis are complications that commonly manifest in patients with cirrhosis. Albumin treatment reduces the severity of these complications, as indicated

One crucial element in the management of liver cirrhosis, especially in the context of complications such as ascites and acute decompensation, is albumin infusion [13]. Figure 1 presents an overview of the role of albumin in resolving the different complications of cirrhosis.

### Albumin-infusion manages paracentesis-induced circulatory dysfunction induced by ascites treatment

Ascites is classified into three grades: mild (grade 1, detectable by ultrasound), moderate (grade 2, mild abdominal distension), and large (grade 3, significant abdominal distension) [14]. The definitive treatment for grade 3 ascites is large volume paracentesis (LVP). This is an invasive procedure where a volume of more than 5 L of ascites is drained from the peritoneal cavity [15]. However, there is the risk of paracentesis-induced circulatory dysfunction (PICD). This is “a complication of large volume paracentesis that leads to a faster re-accumulation of ascites, hyponatremia, renal impairment, and shorter survival” [16]. Hypovolemia caused by a decrease in systemic vascular resistance underpins the development of PICD. Initially, cardiac output increases post paracentesis [17]. This causes haemodynamic shear stress (force of blood flow acting on endothelium), thereby increasing nitric oxide availability [18]. Nitric oxide then causes vasodilation and decreases systemic vascular resistance [19]. It has been theorised that the reduction in intra-abdominal pressure post paracentesis also contributes to the overall reduction in systemic vascular resistance [20]. The resulting effective hypovolemia that occurs due to arterial vasodilation activates the RAAS pathway and sympathetic nervous system [16]. Patients with cirrhosis are unable to fully compensate for their arterial vasodilation effects due their diminished response to the vasoconstrictors. This leads to the symptomatic effects of PICD [21]. PICD is diagnosed as a more than 50% increase in baseline plasma renin levels, reaching > 4 ng/mL/hour, which occurs as a result of commencing LVP without a plasma expander [16, 22].

Albumin can be used to reduce the detrimental effects of ascites treatment and prevent PICD (Fig. 1). The infusion of albumin in cirrhotic patients after LVP treatment causes expansion of intravascular plasma volume. This occurs due to albumin’s effect on colloid osmotic pressure resulting in an extremely quick increase in effective blood volume (total portion of blood volume that actively contributes to perfusion and oxygen delivery to tissues). Resultantly, water moves into the intravascular compartment [23]. This action reverses the hypovolemia observed in PICD.

A meta-analysis highlighted that albumin is superior to any other plasma expander or vasoconstrictor in preventing PICD. Also, it is superior to other plasma volume expanders such as polygeline (synthetic derivative of gelatine which increases plasma volume and improves tissue perfusion). This is because albumin has shown to reduce i) the number of liver-disease-related complications and ii) the time spent in hospital [24].

The recommended dosage for albumin infusion to prevent PICD is 6–8 g/L litre of removed ascites (Table 1). Guidelines also indicate that the albumin should be infused slowly to prevent fluid overload [25]. This is because patients with cirrhosis potentially have latent cirrhotic cardiomyopathy, which is “a combination of reduced cardiac contractility and systolic/diastolic dysfunction” [26]. Cirrhotic patients with advanced cardiomyopathy show a state of fluid overload in the abdomen and in peripheral tissues [27], along with increased capillary permeability, as seen in cirrhosis [28]. Portosystemic shunts found in cirrhosis patients can worsen cardiac function due to an extremely rapid shift of blood from the splanchnic circulation to the heart after albumin infusion [29].

**Table 1:** Albumin dosage for cirrhosis complications as per Caraceni et al. and Campos et al. [58, 59]

Cirrhosis complications	Percentage of albumin concentration	Dosage of albumin to be infused
PICD	20–25%	6–8 g albumin per 1L fluid removed
Type 1 HRS	20–25%	1 g/kg of albumin for 2 days. 20-40 g/day on day 3 onwards
SBP	20–25%	1.5 g/kg of albumin on day 1 and 1 g/kg on day 3

### Albumin-diuretics combination for ascites

Albumin can be used in combination with diuretics in the treatment of ascites (Fig. 1). Diuretics such as spironolactone (aldosterone antagonist) is commonly used in isolation to increase the excretion of sodium and water. When combined with albumin, there is a marked and significantly increased response to the diuretics, thereby improving ascitic fluid drainage. One study compared two groups of patients with liver cirrhosis: one group receiving diuretic and the other group receiving diuretics as well as albumin. The rate of response to treatment was higher in the group receiving albumin, and their hospital stay was significantly shorter (20 ± 1 versus 24 ± 2 days) [30].

### Albumin in the treatment of spontaneous bacterial peritonitis and prevention of hepatorenal syndrome, the complications of liver cirrhosis

Spontaneous bacterial peritonitis (SBP) can occur in cirrhotic patients with ascites. Here, there is an infection of the ascitic fluid in the peritoneal cavity without a clearly identifiable source of infection [31]. SBP can progress and drive further complications such as hepatorenal syndrome, haemorrhage and hypovolemic hyponatremia [32]. The pathophysiology of SBP is unclear. However, it is considered to involve the movement of bacteria and endotoxins from the gastrointestinal tract to the peritoneal fluid [33]. This bacterial movement is supported by an impairment of the intestinal barrier and reduced bowel motility of patients with cirrhosis, which increases intestinal transmit time [34]. Moreover, cirrhosis patients show poor phagocytic and reticuloendothelial system function [35]. This results in difficulty in clearing bacteria and endotoxins in SBP in these patients. Thus, bacteria are able to proliferate and migrate to the ascitic fluid [36]. These bacterial endotoxins and cytokines collectively cause a marked increase in the production of nitric oxide in patients with cirrhosis [37]. Nitric oxide’s vasodilator effects result in worsened arterial vasodilation leading to hypoperfusion of the kidneys [38]. This phenomenon is hepatorenal syndrome (explained later) and is a direct consequence of SBP.

In addition to bacterial presence, high levels of cytokines such as tumour necrosis factor α (TNFα), interleukin-6 (IL-6) and interleukin-1(IL-1) can commonly be detected in cirrhotic patients with SBP, which triggers sepsis syndrome pathways and eventually lead to renal failure [39].

Circulatory dysfunction and renal impairment are the main complications observed in patients with SBP. Low mean arterial pressure (MAP) (average arterial pressure throughout one cardiac cycle) has been shown to be correlated with higher mortality in cases of kidney dysfunction associated with cirrhosis [40]. A reduced MAP has also been linked with worsening of hepatorenal syndrome [41].

Albumin acts as a treatment facilitator in patients with SBP (Fig. 1). For example, intravenous albumin has been shown to significantly increase MAP through its potent plasma expander qualities, which is vital for organ perfusion [42]. Also, administration of albumin has shown to decrease the heart rate and reduce RAAS pathway activation, thereby improving the circulatory dysfunction seen in SBP and reducing renal impairment [42]. In a study conducted by Chen et al., albumin displayed a capacity to bind to inflammatory cytokines such as IL-6 and TNFα and reduce their effect in the ascitic fluid, when combined with antibiotics [43]. In another retrospective cohort study, incidences of acute kidney injuries and fatality were significantly lower in cirrhotic SBP patients treated with albumin in combination with antibiotics. The incidences of acute kidney injuries were 63.93% in patients treated with antibiotics only and 33.33% in patients treated with a combination of antibiotics and albumin [44]. Unsurprisingly, albumin is associated with decreased mortality in patients with SBP. A meta-analysis indicated a 19.4% difference in mortality between patients treated with and without albumin. Multiple clinical trials concluded that albumin infusion significantly reduced incidences of renal impairment and decreased mortality [45].

Human albumin is indicated to be infused at 1.5 g per kilogram of bodyweight upon diagnosis of SBP and then 1 g per kilogram of bodyweight on the third day. Serum creatinine and bilirubin levels of these patients with SBP also need to be monitored. If creatinine is < 1 mg/dL and bilirubin is < 4 mg/dL, then albumin infusion may be unnecessary because this group of cirrhosis patients are at a low risk of developing severe complications like renal failure [25] (Table 1).

### Albumin in the treatment of hepatorenal syndrome (HRS)

SBP can precede the development of hepatorenal syndrome (HRS) in patients with cirrhosis. HRS is a life threatening, multiorgan condition of renal impairment observed in patients with decompensated liver cirrhosis [46]. HRS is of 2 types. Type 1 is a rapid and progressive impairment of renal function. Here, serum creatinine rises to a level higher than 2.5 mg/dL (221 μmol/L) in less than 2 weeks. Type 2 is less severe than type 1 and presents with initial creatinine levels below 2.5 mg/dL (221 μmol/L) [47]. The survival time for patients with type 1 HRS following the onset of renal failure is 8–10 weeks. The survival time  is 6 months for patients with type 2 HRS [48].

**Fig. 2:**
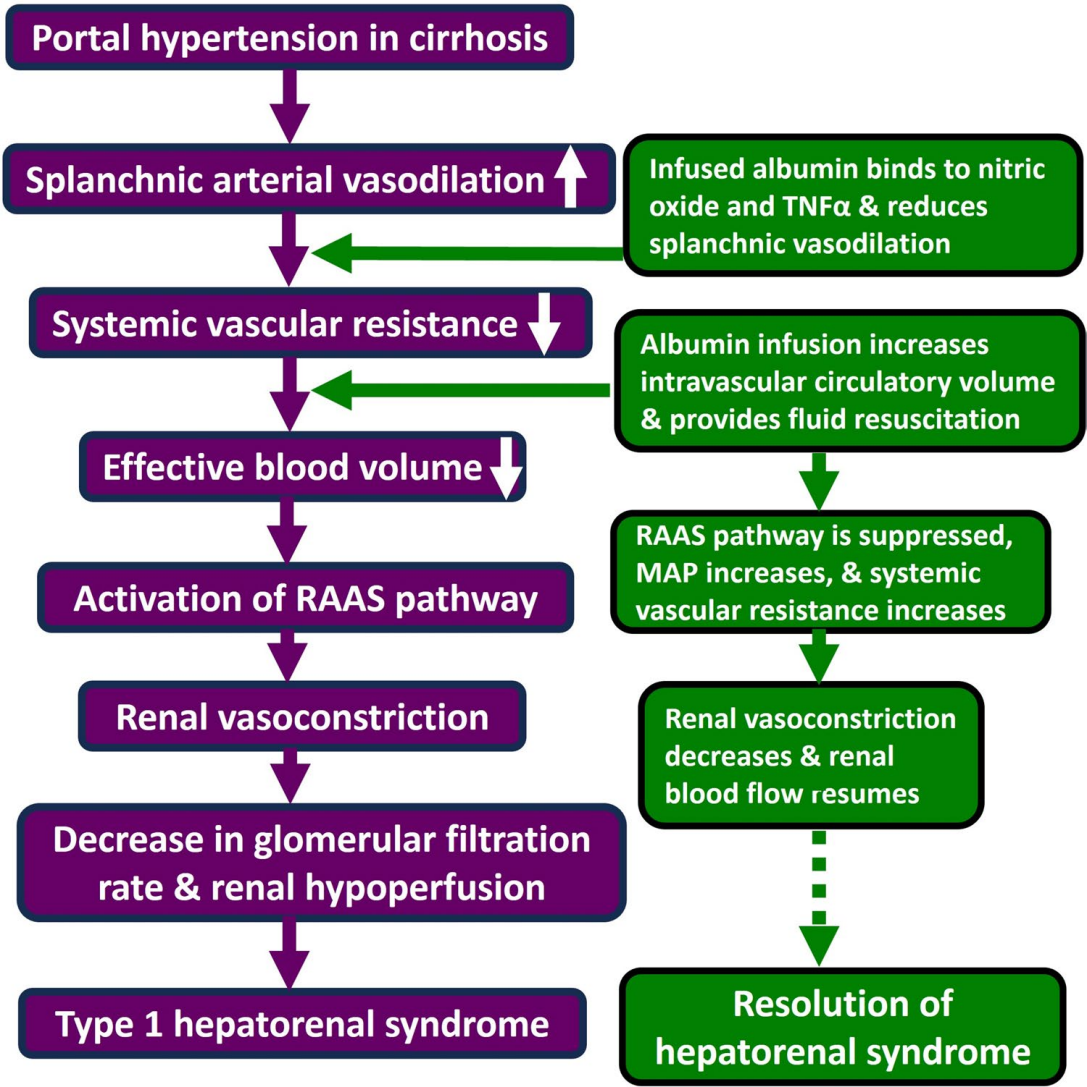
**Development of type-1 hepatorenal syndrome (HRS) in cirrhotic patients and the role of albumin infusion.** The figure illustrates progressive renal dysfunction in cirrhotic patients due to haemodynamic alterations including splanchnic vasodilation, decreased effective arterial blood volume, and then activation of the RAAS pathway. These events lead to renal vasoconstriction, reduced glomerular filtration rate, and ultimately contribute to the development of HRS. Albumin infusion (in combination with a vasoconstrictor) can reduce splanchnic vasodilatation, supress RAAS pathway, reduce renal vasoconstriction, and thereby help resolve HRS in about 50% of patients [52]

As depicted in Fig. 2, portal hypertension precedes the physiological changes that occur in patients with HRS [46]. Arterial splanchnic vasodilators such as nitric oxide, carbon monoxide and cyclooxygenase-derivatives are released in the early stages of liver cirrhosis in response to portal hypertension [10]. In turn, arterial vasodilation leads to an underfilling of the systemic arterial vascular space. This leads to a drop in arterial blood pressure and a decrease in effective blood volume [49]. This is followed by the activation of the RAAS pathway and release of vasoconstrictive agents such as aldosterone and endothelin [50]. In the early stages of decompensated liver cirrhosis, this would not affect renal perfusion because opposing vasodilating factors such as prostaglandins are synthesised and released [51]. However, as decompensated liver cirrhosis progresses, renal perfusion cannot be maintained as there is an aggressive activation of vasoconstrictive systems. This hypoperfusion of the kidneys combined with decreased production of vasodilators leads to the development of HRS [46] (Fig. 2).

The figure illustrates progressive renal dysfunction in cirrhotic patients due to haemodynamic alterations including splanchnic vasodilation, decreased effective arterial blood volume, and then activation of the RAAS pathway. These events lead to renal vasoconstriction, reduced glomerular filtration rate, and ultimately contribute to the development of HRS. Albumin infusion (in combination with a vasoconstrictor) can reduce splanchnic vasodilatation, supress RAAS pathway, reduce renal vasoconstriction, and thereby help resolve HRS in about 50% of patients [52].

Albumin infusion is vital for treating HRS. One way in which this occurs is that albumin provides small volume fluid resuscitation [52]. Albumin’s oncotic properties facilitate the redistribution of a part of the splanchnic circulation into the central circulatory volume. A study highlighted that albumin increased intravascular circulatory volume by around 210–260% [53]. Increasing intravascular volume in this way is preferred to aggressive fluid infusion in patients with type 1 HRS. This is because they are at higher risk of worsening ascites and pleural effusion due to their substantial fluid load [54]. Moreover, albumin infusion, when combined with vasoconstrictors like midodrine and octreotide, has shown to significantly reduce renin and aldosterone concentrations [55], which leads to reduction in renal vasoconstriction (Fig. 2).

Albumin’s anti-inflammatory properties are another asset in treating HRS. Nitric oxide, one of the potent splanchnic vasodilators that contributes to the development of HRS, can be bound to albumin. TNFα concentrations were also significantly reduced when albumin was administered [43] (Fig. 2). Studies have indicated that albumin may have an impact on cardiac function impairment in patients with HRS. One study conducted on cirrhotic rats showed that cardiac contractility was significantly increased by albumin infusion. There was also evidence of albumin directly counteracting oxidative stress and interfering with TNFα pathways that impaired cardiac contractility [56].

There is a treatment plan in combination with vasoconstrictive agents such as terlipressin. In this plan, albumin is infused along with terlipressin at a dosage of 1 g/kg of body weight on the first day of treatment upon diagnosis of HRS. This is followed by 20–40 g of albumin infusion per day until terlipressin is stopped or for a maximum of 2 weeks. The albumin dosage is also reduced/stopped if pulmonary oedema develops following the albumin treatment [25, 57] (Table 1).

### Overview of albumin dosage in cirrhosis-associated complications

Albumin dosages for patients with cirrhosis vary based on specific clinical needs and conditions. Table 1 presents a comparative overview of recommended albumin dosages used in the management of cirrhosis-associated complications such as ascites, hepatorenal syndrome, and spontaneous bacterial peritonitis. Dosages may vary depending on individual patient factors and treatment protocols.

### Putative role of albumin infusion in hepatic encephalopathy (HE)

Ammonia is primarily formed from nitrogenous products in the diet, metabolism of urea and from the deamination of glutamine in the small intestine [60]. Typically, the liver converts ammonia into urea, which is then excreted by the kidneys. However, due to hepatocyte malfunction in cirrhosis and the development of portosystemic collaterals (abnormal communications between portal system and systemic circulation), ammonia clearance becomes insufficient [61]. Consequently, the concentration of ammonia in the bloodstream increases, and it can breach the blood–brain barrier, potentially causing brain oedema [62]. Also, in patients with decompensated cirrhosis, there is a marked increase in proinflammatory cytokines such TNFα and IL-6 [63]. These cytokines have the potential to compromise the integrity of the blood–brain barrier and facilitate the movement of ammonia ions into the brain, resulting in symptomatic or overt hepatic encephalopathy [64].

Essentially, hepatic encephalopathy (HE) is the result of accumulation of neurotoxic substances in the brain such as ammonia, mercaptans, GABA and short chain fatty acids [65]. This accumulation causes neurological symptoms such as confusion, personality alterations and disorientation [65]. HE is commonly associated with liver failure, and it is a negative prognostic indicator for cirrhosis patients. Cirrhosis patients with HE show a survival rate of only 23% after 3 years from onset [66].

Free radicals cause cerebral oedema in HE. Reactive oxygen and nitrogen species damage astrocytes by triggering RNA and protein modifications [67]. This damage occurs in the presence of ammonia and pro-inflammatory markers, which eventually increases the production of glutamine by astrocytes. Glutamine increases osmotic pressure, resulting in swelling of the astrocytes. This swelling in referred to as the cerebral oedema observed in HE and causes some of the HE-associated symptoms [68].

Albumin infusion has shown the potential to treat HE and reduce its complications. For example, its potent plasma expander qualities can reduce the levels of ammonia in plasma by increasing urinary ammonia excretion [69]. Also, albumin’s unique structure (with a free cysteine-34 residue) can ameliorate inflammation by binding to TNF and IL-6, thereby preventing the breach of the blood–brain barrier [70]. Furthermore, albumin also displays potent antioxidant effects which can be vital in treating HE. For example, ions of copper and iron form free radicals that contribute to  the development of oedema. Albumin binds to these metal ions and exhibit a protective effect [71]. Therefore, albumin infusion should be able to ameliorate the condition in patients with overt HE. Indeed, some studies have shown beneficial effects of albumin in treating HE. For example, in a clinical trial, when patients with diuretic-induced-HE were treated with albumin, they showed a significant improvement in their mental status. There was also a marked reduction in oxidative stress markers [69]. Sharma et al. administered albumin with lactulose (laxative) and observed a decrement in the levels of ammonia, reduction in mortality and reversal of HE [72]. However, other studies have shown no effect of albumin [73].

### Critical evaluation of albumin treatment for cirrhosis-associated complications

Historically, the use of albumin has been mired in controversy. This is because the hyper-oncotic nature of albumin has the potential to cause adverse effects in patients with decompensated cirrhosis. For example, there is increased capillary leakage in critically ill patients and there is the risk of albumin leaking into the interstitial space [74]. This leakage can lead to a fourfold increase in fluid retention causing pulmonary oedema [75]. There is also the risk of renal impairment in cirrhotic patients being treated with albumin. This is due to a decrease in glomerular filtration rate following albumin infusion [76]. The cause of this decrease is not known; however, it could be due a decrease in albumin clearance. These albumin aggregates could then prevent filtration from the glomerulus [77]. More recently, in rat models, human albumin infusion has also been shown to increase intercranial pressure by increasing the permeability of the blood–brain barrier [78]. In another study, albumin infusions failed to consistently improve circulatory function in patients with cirrhosis and ascites [79]. This could be due to albumin escaping into the interstitial space, and therefore unable to function as plasma expander. However, it has been postulated that albumin infusion alone is unable to efficiently increase effective arterial blood volume due to the major vasodilation in the splanchnic circulation [80]. This is due to the activation of vasodilators such as nitric oxide and cyclooxygenase derivatives in response to portal hypertension [10]. Notably, there are no effective results indicating that short-term albumin infusion can reduce mortality in patients with decompensated cirrhosis that present with different complications [81].

On the other hand, multiple case studies on the efficacy of albumin infusion in cirrhotic patients have indicated that albumin infusion has positive effects in patients with type 1 HRS, SBP and PICD. These 3 complications are linked by their common pathophysiological origin of effective hypovolemia and the requirement for fluid resuscitation [82].

The ANSWER clinical trial which investigated the long-term administration of human albumin in patients with decompensated cirrhosis showed improvements in hemodynamic stability, renal function, and overall survival [83]. In this study, 431 patients with grade 2 and 3 ascites were administered with 40 g of albumin twice a week initially, and then 40 g once a week after the initial period. Refractory ascites and the requirement for LVP treatment to drain ascites reduced by 50%. There was also a reduction in SBP, type 1 HRS and HE by 30–67%. Importantly, there was a 45% reduction in the number of days spent in hospital for patients with cirrhosis and a 38% reduction in mortality. Serum concentration of albumin at 4 g/dL was identified as a cutoff point to predict survival because this correlated with the probability of 18-month survival. Although the ANSWER trial indicated that long-term administration of albumin improved survival in decompensated cirrhosis, the trial had some limitations. Primarily, the open label design of the trial meant that there was no blinding. Also, placebo control was not set up and the patients with albumin infusions were seen more frequently by healthcare professionals, which introduced an inherent bias [83].

The ATTIRE study included 777 patients with decompensated cirrhosis, all with albumin levels < 30 g/L. For the targeted albumin treatment group, there was a median total infusion of 200 g albumin for up to 14 days or until discharge. The median total infusion for the standard-care group (albumin only administered if they had HRS, LPV or SBP) was 20 g. The study evaluated the incidence of primary end point events. These included a new infection, kidney dysfunction or death between days 3 and 15 after the treatment begun. Results showed that the percentage of patients with a primary end point event was not significantly different between both groups (29.7% in the targeted albumin treatment group and 30.2% in the standard care group). The targeted albumin treatment group showed more severe or life-threatening complications due to the treatment, including the development of pulmonary oedema and multiorgan failure [81]. These results show that albumin treatment can help some patients with PICD, SBP and HRS. However, it cannot be justified as a treatment for all patients with advanced cirrhosis.

The MACHT trial was another large-scale randomised control trial conducted to examine the efficacy of albumin infusions. It included 196 patients with decompensated cirrhosis and ascites. These patients were awaiting liver transplantation. The test group receiving human albumin infusion plus midodrine (vasoconstrictor) was compared to a placebo control group. The aim of the trial was to ameliorate the cirrhosis-induced circulatory dysfunction. The dosage of albumin was 40 g of 20% albumin for 15 days or until there was a primary end-point event (renal failure, hyponatremia, infections, hepatic encephalopathy, or gastrointestinal bleeding). As with the ATTIRE trial, there was no significant difference between both groups in i) the progression of cirrhosis complications and ii) one-year mortality rate. However, there was an impact on the RAAS pathway. There was a significant decrease in plasma renin activity and aldosterone levels compared to placebo [84]. This trial, like the ATTIRE study, also had limitations. For example, many treatment pathways were interrupted before they had been completed due to patients’ liver transplantations. As a result, less than 10 patients were able to complete their course of albumin infusion.

### Cirrhosis-induced modifications of native albumin and its utilisation in prognosis

Cirrhosis induces elevation in serum concentration of pro-oxidant and proinflammatory substances [85]. This leads to post-transcriptional changes to albumin via glycosylation or oxidation that target the free cysteine-34 position on albumin [86]. Diabetes is prevalent in patients with cirrhosis and hyperglycaemia-induced physiological stress adds to this damage to albumin. Determination of these cirrhosis-induced structural alterations in albumin has shown prognostic value. For instance, the altered forms of albumin have been thought to act as a biomarker to predict patient outcomes with acute decompensated cirrhosis. In addition, these altered forms of albumin can indicate progression into other complications of cirrhosis such as HRS and SBP and allow for more prompt treatment initiation [87].

A study investigated on the prevalence of cirrhosis-induced post-transcriptional changes to albumin and how this affected cirrhotic patient outcomes. 168 patients with cirrhosis and 94 healthy controls were enrolled into the study. Patients then had their serum albumin isoforms analysed and compared with the control group. Here, multiple human serum albumin isoforms were identified including Human Serum Albumin (HSA)-DA (truncation of last two amino acids residue), HSA + CYS (Cysteinylation of Cys-34 residue) and HSA + GLYC (glycosylation of Cys-34 residue). The relative concentration of unaltered native albumin was significantly lower in patients with cirrhosis. Moreover, all albumin alterations that impacted the free cysteine-34 residue directly correlated with a higher MELD (model for end stage liver disease) score and Child–Pugh score. In patients with ascites, there were significantly increased levels of HSA-DA (N-terminal truncated) and HSA + CYS-DA, along with significantly lower levels of the native HSA isoform. This study confirmed the presence of HSA + CYS-DA (cysteinylated and N-terminal truncated albumin) only in patients with ascites. Patients with cirrhosis and SBP showed higher levels of HSA-L (C-terminal truncated albumin) and HSA + CYS-DA [85]. Moreover, a higher concentration of native serum albumin was associated with an increase in one-year survival [88]. Thus, this study indicated that altered isoforms of human albumin could be utilised to ascertain cirrhosis-induced complications. The utilisation of effective serum albumin (i.e., functional albumin levels) as a prognostic factor is also justified by this study.

The structural integrity of albumin plays a very important role in cirrhosis patients because of albumin's anti-inflammatory and antioxidant functions. Albumin isoforms show reduced binding constant compared to native albumin [89]. So, it is possible that cirrhosis-induced alterations in the native albumin incapacitates the binding of albumin to toxins and pro-inflammatory cytokines, thereby hampering its usual anti-inflammatory functions.

Currently, total serum albumin is utilised to understand the progression of cirrhosis severity [90]. However, effective serum albumin has been found to be a more insightful prognostic marker because it declines with increased cirrhosis severity [90, 91]. A decrease in effective serum albumin is also associated with an increase in proinflammatory cytokines [91], which in turn is linked to worsening decompensation of cirrhosis [92]. Baldassare et al. aimed to compare the utility of effective and total serum albumin in evaluating cirrhosis-induced complications. They observed that the decrease in effective serum albumin was greater than the decrease in total serum albumin in cirrhosis patients with SBP and renal failure [91].

## Summary

Liver cirrhosis can emerge from various aetiologies. It is a complex and often irreversible condition. It shows progression from the asymptomatic stages to the symptomatic decompensated stages and is associated with severe complications in its advanced stages. Albumin is a major blood protein synthesised by the liver. When it is administered in cirrhosis patients, it can play a crucial role in managing these complications. Albumin infusion can regulate fluid balance, effectively treat ascites, prevent paracentesis-induced circulatory dysfunction (PICD), and aid in managing hepatorenal syndrome (HRS) and spontaneous bacterial peritonitis (SBP). The multifaceted contributions of albumin infusion in cirrhosis underline its potential in improving patient outcomes and addressing the challenges associated with cirrhosis management. However, albumin infusion is neither applicable nor effective for all patients.

## Data Availability

Not applicable.
